# Predictors of Response to Ketamine in Treatment Resistant Major Depressive Disorder and Bipolar Disorder

**DOI:** 10.3390/ijerph15040771

**Published:** 2018-04-17

**Authors:** Carola Rong, Caroline Park, Joshua D. Rosenblat, Mehala Subramaniapillai, Hannah Zuckerman, Dominika Fus, Yena L. Lee, Zihang Pan, Elisa Brietzke, Rodrigo B. Mansur, Danielle S. Cha, Leanna M. W. Lui, Roger S. McIntyre

**Affiliations:** 1 Mood Disorders Psychopharmacology Unit (MDPU), University Health Network, University of Toronto, Toronto, ON MT5 2S8, Canada; carola.rong@gmail.com (C.R.); caroline.park@mail.utoronto.ca (C.P.); joshua.rosenblat@uhn.ca (J.D.R.); mehala.subram@uhn.ca (M.S.); hannahzuckerman2@gmail.com (H.Z.); dominikafus@icloud.com (D.F.); yenalee.lee@mail.utoronto.ca (Y.L.L.); danny.pan@mail.utoronto.ca (Z.P.); elisabrietzke@hotmail.com (E.B.); rodrigomansur71@uol.com.br (R.B.M.); cha.danielle@gmail.com (D.S.C.); leanna.lui19@gmail.com (L.M.W.L.); 2 Institute of Medical Science, University of Toronto, Toronto, ON M5S 1A8, Canada; 3 Department of Psychiatry, University of Toronto, Toronto, ON M5T 1R8, Canada; 4 Department of Psychiatry, Federal University of São Paulo, São Paulo 05403-903, Brazil; 5 Department of Pharmacology, University of Toronto, 399 Bathurst Street, Toronto, ON M5T 2S8, Canada

**Keywords:** ketamine, depression, MDD, response, predictors, remission

## Abstract

Objectives: Extant evidence indicates that ketamine exerts rapid antidepressant effects in treatment-resistant depressive (TRD) symptoms as a part of major depressive disorder (MDD) and bipolar disorder (BD). The identification of depressed sub-populations that are more likely to benefit from ketamine treatment remains a priority. In keeping with this view, the present narrative review aims to identify the pretreatment predictors of response to ketamine in TRD as part of MDD and BD. Method: Electronic search engines PubMed/MEDLINE, ClinicalTrials.gov, and Scopus were searched for relevant articles from inception to January 2018. The search term *ketamine* was cross-referenced with the terms *depression, major depressive disorder, bipolar disorder, predictors*, and *response* and/or *remission*. Results: Multiple baseline pretreatment predictors of response were identified, including clinical (i.e., Body Mass Index (BMI), history of suicide, family history of alcohol use disorder), peripheral biochemistry (i.e., adiponectin levels, vitamin B12 levels), polysomnography (abnormalities in delta sleep ratio), neurochemistry (i.e., glutamine/glutamate ratio), neuroimaging (i.e., anterior cingulate cortex activity), genetic variation (i.e., Val66Met BDNF allele), and cognitive functioning (i.e., processing speed). High BMI and a positive family history of alcohol use disorder were the most replicated predictors. Conclusions: A pheno-biotype of depression more, or less likely, to benefit with ketamine treatment is far from complete. Notwithstanding, metabolic-inflammatory alterations are emerging as possible pretreatment response predictors of depressive symptom improvement, most notably being cognitive impairment. Sophisticated data-driven computational methods that are iterative and agnostic are more likely to provide actionable baseline pretreatment predictive information.

## 1. Introduction

Major Depressive Disorder (MDD) is a highly prevalent, chronic, and disabling disorder that is affecting approximately 350 million individuals worldwide, with only one-third of affected individuals remitting with first-line antidepressant treatment [1] Moreover, approximately one-third of individuals fail to achieve syndromal and functional recovery despite receiving multimodality treatment interventions [2]. A modifiable deficiency in the management of MDD is in reducing the time to onset of clinically significant improvement in symptoms with conventional treatment interventions (e.g., antidepressant medication). For example, it often requires approximately 4 to 8 weeks of treatment before clinically significant therapeutic improvements are observed [3]. This therapeutic inertia prolongs the patient-reported symptoms of depression, leaves individuals at a greater risk for suicidality, and increases the economic costs (direct and indirect) that are related to MDD [4].

Convergent evidence indicates that subanesthetic doses of ketamine, which is a dissociative anesthetic that antagonizes glutamatergic N-methyl-D-aspartate (NMDA) receptors, has rapid-onset antidepressant effects in subpopulations with MDD and bipolar disorder (BD) that do not sufficiently respond to conventional antidepressant therapies [5]. In addition to antidepressant effects, anti-suicide effects have also been reported with ketamine, independent of its salutary effects on total depressive symptom severity [6,7,8]. It has been reported that the magnitude of therapeutic benefits that are associated with ketamine treatment in treatment resistant depression (TRD) is comparable to the therapeutic benefits that are associated with electroconvulsive therapy [9]. However, the current evidence is not sufficient to recommend ketamine as an antidepressant, as ketamine may carry the risk of diversion and misuse. The medical communities should conduct more research and be more critical of the evidence provided by ketamine trials [10].

The mechanism of action of ketamine has not yet been fully characterized, but is hypothesized to involve downstream actions on multiple effector systems, including, but not limited to, the monoamine system, the opioid system, the gamma aminobutyric acid (GABA)/glutamate system, signal transduction cascades (i.e., mTor, rapamycin), cellular proliferation, and neuroplasticity-promoting intracellular cascades [11,12]. A single subanesthetic (0.5 mg/kg) intravenous (IV) infusion of ketamine over 40 min has been demonstrated to result in significant improvements of depressive symptoms within 4 h, and lasting up to seven days [5]. After infusion of ketamine, the response rate at 6 h is 54%; day 1 is 71%; and, day 3 is 54% [13]. The mean duration to relapse of another depressive episode is 17.2 days after a single infusion of ketamine. The antidepressant effects of ketamine in treatment-resistant mood populations have been supported by multiple lines of evidence, including original research, systematic reviews, and meta-analyses [14].

A primary clinical concern related to the therapeutic use of ketamine for TRD is tolerability (e.g., dissociative phenomena, vasomotor effects) and safety (e.g., CNS effects) concerns, with both short- and long-term administration. There is a lack of studies evaluating the systemic side effects of repeated IV ketamine infusion, including cystitis and urinary incontinence [15]. The need for IV infusion and post-administration surveillance with IV ketamine introduces complexities and costs that are inherent to ketamine treatment [16]. Hence, the identification of pretreatment predictors of safety, tolerability, and efficacy in response to IV ketamine would provide the opportunity to empirically select (and exclude) subpopulations with TRD who are more likely (or less likely) to benefit from treatment. In addition to improving therapeutic response prediction, delineating subpopulations with TRD that are more likely to benefit from ketamine treatment has implications for cost-modeling cost-effectiveness analyses.

Herein, we aim to identify and summarize pretreatment clinical and biological predictors of successful response to IV ketamine infusion in adults with TRD as part of MDD and BD. We purposefully delimit our focus to reports evaluating IV ketamine infusion, as this is the most commonly reported delivery method in the biomedical literature.

## 2. Methods

An electronic literature search was conducted using the following databases: PubMed/MEDLINE, ClinicalTrials.gov, and Scopus from inception to January 2018. The search term *ketamine* was cross-referenced with the terms *depression, major depressive disorder, bipolar disorder, predictors*, and *response* and/or *remission*. All of the identified articles were screened for inclusion in the present review based on their title and abstract. Search results that were irrelevant to the scope of this manuscript were excluded from further review. Articles were manually reviewed with an aim to identify variables that were of focus in the review herein. Bibliographies were also manually searched to identify additional pertinent studies. Inclusion criteria were established prior to article review and were as follows:

1. adult subjects (ages 18–65) with a diagnosis of MDD or BD and/or TRD as defined by the Diagnostic and Statistical Manual (DSM);
2. study was an intervention trial of IV ketamine infusion; and,
3. depression severity was assessed before and after ketamine infusion, and reported using standardized and validated depression rating scales.

Exclusion criteria were:

1. unpublished data;
2. preclinical studies; and,
3. reports on duplicated datasets.

## 3. Results

The original search yielded a total of 582 records; 511 records were excluded that were not human clinical trials (i.e., preclinical trials). After the screening of titles and abstracts, 12 studies evaluating response to ketamine in adults with MDD or BD and/or TRD were included in the review; nine studies were included via electronic search; and, three studies were included via manual search. Results from the literature search are summarized in Table 1.

### 3.1. Sociodemographic Variables

No sociodemographic factors were found to robustly predict patient response to ketamine.

### 3.2. Clinical Variables

A positive family history of alcohol use disorder (FHP) was associated with a greater probability of response to ketamine infusion in individuals with MDD and BD when compared to healthy controls, as evidenced by the change in total Montgomery-Asberg Depression Scale (MADRS) score from baseline to endpoint [17,18]. Subjects with a positive family history of alcohol use disorder exhibited greater improvement in Hamilton Depression Rating Scale (HDRS) score at one-day and seven-days post-infusion, but not at 230 min post-infusion. A study that was conducted by Niciu et al. (2014) expanded on this finding by demonstrating that the antidepressant effects of ketamine could be sustained for up to four weeks in subjects with a FHP [19].

In addition to FHP, a separate post-hoc analysis that was conducted by Niciu et al. (2014) reported that a symptomatic improvement in MDD and BD subjects (*n* = 108) was moderated by body mass index (BMI) and a prior history of suicide attempts. Specifically, higher pretreatment BMI was correlated with greater improvement in total HDRS score at 230-min and one-day post-infusion, but not at seven-days post-infusion. A meta-analysis has established a positive association between obesity and early-onset depression [27]. Epidemiological studies found that increased BMI was associated with depressive symptoms in adults [28]. Stressors can cause down-regulation of leptin signalling, resulting in poor appetite control [29].

In addition, individuals without a history of suicide attempts had greater improvement in HDRS score at seven-days post-infusion, but not at earlier time points. Moreover, BMI was associated with acute symptomatic improvement, while FHP and no prior history of suicide attempts were associated with a sustained antidepressant response to ketamine [30]. It was also reported that while higher BMI as a pretreatment response predictor to ketamine might possibly be an epiphenomenon of a relatively higher administered dose of ketamine in this subpopulation.

### 3.3. Peripheral Biomarkers

Machado-Vieira et al. (2017) sought to identify specific adipokines that moderate the relationship between IV ketamine response, anthropometrics (i.e., BMI), and depressive symptomatology. They observed that low pretreatment plasma adiponectin levels predicted rapid response to ketamine [20]. Adiponectin is an adipokine (i.e., cytokine secreted by adipose tissue) that is known to exert anti-inflammatory and insulin-sensitizing effects. The observation of pretreatment pro-inflammatory balance may be a potential predictor of an antidepressant response. The levels of pro-inflammatory cytokines such as interleukin-6 (IL-6) and tumor necrosis factor (TNF-α) have been reported to be higher in MDD subjects than controls [31]. Conventional antidepressants (e.g., fluoxetine) have been found to reduce both central and peripheral levels of the pro-inflammatory cytokine interleukin-1 beta (IL-β) in the alleviation of depressive symptoms [32]. Other treatments with antidepressant properties have also been reported with mechanistically dissimilar treatments (e.g., omega 3 fatty acids, L-methylfolate) [33].

In addition to pretreatment pro-inflammatory cytokines potentially moderating response to ketamine, preliminary evidence indicates that circulating vitamin B12 levels may affect the treatment outcomes with ketamine [34]. Previous reports indicate that higher levels of circulating vitamin B12 are associated with a greater probability of response to conventional antidepressants [35]. 

To contextualize the foregoing results, a single small study with 20 subjects identified vitamin B12 levels were cross-sectionally associated with bipolar depression. Specifically, ketamine treatment “responders” had higher levels of circulating vitamin B12 when compared to “non-responders”, where “responders” were subjects with a 50% or greater reduction of HDRS, as compared to baseline, on the seventh day after infusion. This result is consistent with studies showing that higher levels of vitamin B12 are positively correlated with the conventional antidepressant response [21,35].

### 3.4. Polysomnography

Slow wave sleep has consistently been reported to be decreased in individuals with MDD and BD [36]. Delta sleep ratio (DSR) is defined as the ratio of slow wave activity between the first two non-REM sleep episodes, and it is reported to be relatively lower in MDD patients than in healthy controls [37]. In a study by Duncan et al. (2013), it was observed in 30 subjects with MDD that low pretreatment DSR was associated with a greater improvement in total depressive symptom severity, whereas high pretreatment DSR predicted a less favourable response to IV ketamine treatment [22].

### 3.5. Neurochemistry Variables

Abnormalities in amino acid neurotransmitter systems are postulated to play a critical role in the pathoetiology of MDD [38]. Stress-induced depression and cognitive impairment have been found to be associated with the reduced expression of the gamma-aminobutyric acid (GABA) receptors in the brain [39].

Using proton magnetic resonance spectroscopy (^1^H-MRS), Salvadore et al. (2012) measured levels of the amino acid neurotransmitters gamma aminobutyric acid (GABA), glutamate, and Glx/glutamate (a surrogate marker of glutamine), in the ventromedial and the dorsomedial/dorsal anterolateral prefrontal cortex before and after IV ketamine treatment in subjects with MDD (*n* = 14). Following ketamine infusion, depressive symptoms significantly improved after 230 min, as assessed by the changes in the mean MADRS score. The authors reported that while pretreatment GABA and glutamate did not correlate with an improvement of depressive symptoms, pretreatment Glx/glutamate ratio was found to be significantly and inversely correlated with the symptomatic improvement with ketamine [23].

### 3.6. Noninvasive Functional Neuroimaging

Salvadore et al. (2009) evaluated the effect of IV ketamine on anterior cingulate cortical (ACC) activity in response to an emotional stimulus in subjects with MDD (*n* = 11) versus healthy controls (*n* = 11). Subjects with MDD exhibited exaggerated ACC activity, which was positively and significantly correlated with a rapid antidepressant response to IV ketamine [24]. The foregoing results suggest that IV ketamine treatment may target neural structures subserving affective processing and cognitive-emotional reactivity. It is hypothesized that these observed effects of ketamine on neural structures and circuits that are relevant to psychopathology in mood disorders is principally mediated by ketamine-induced effects on AMPA-mediated synaptic plasticity [40].

### 3.7. Genetics

Central and peripheral brain derived neurotrophic factor (BDNF) levels are reported to be decreased in individuals with MDD and BD, and are positively correlated with the conventional antidepressant treatment response [41]. In accordance with this, the BDNF Val66Met single nucleotide polymorphism (SNP) has been reported to be associated with an impaired function of BDNF activity in the human brain [42]. Laje et al. (2012) investigated whether the Val66Met SNP was associated with ketamine treatment response in patients with MDD. They reported that the overall mean change in HDRS score from baseline to endpoint was significantly lower in Met carriers when compared to Val carriers. This observation suggests an increased likelihood of response to IV ketamine in depressed individuals carrying the Val/Val BDNF allele at rs6265e [25].

### 3.8. Cognitive Function

Neurocognitive impairment in individuals with MDD and BD is a highly replicated finding and enduring abnormality during and between episodes [43]. Specific symptoms of depression, such as insomnia, are associated with neurocognitive impairment [44]. In addition to serving as a proximate mediator of functional outcome in mood disorders, early improvement in cognitive performance has been suggested as a predictor of treatment response [45]. Preliminary evidence suggests that pretreatment cognitive performance may influence the overall treatment response to ketamine. For example, Murrough et al. (2013) assessed baseline neurocognitive functioning in subjects (*n* = 25) with TRD, using a comprehensive battery (estimated premorbid IQ, current IQ, and tests from the MATRICS battery). At 40 min post-infusion, the participants repeated a subset of the neurocognitive tests, while a change in depression severity was measured using MADRS, with response status being defined as a ≥50% reduction in MADRS score at 24 h relative to baseline. The authors of the study reported that lower levels of baseline neurocognitive performance (in particular processing speed) in individuals with TRD are associated with an increased antidepressant response rate to ketamine [26].

Of note, a separate hypothesis has been proffered which suggests that the pro-cognitive effects of ketamine mediate the anti-suicidal effects that are associated with ketamine treatment in adults with mood disorders [6].

## 4. Discussion

There is an urgent need to identify predictors of response to psychotropic medications in subpopulations with mood disorders. When considering IV ketamine for TRD, the safety, tolerability, and costs associated with this treatment further amplify the need to identify reliable predictors of ketamine response. The identification of pretreatment predictors would guide clinicians and provide decision support as it regards the selection and sequencing of ketamine treatment in a cost-effective manner. Mechanistically, it may be surmised that the variables that predict the ketamine response reflect the putative mechanism of action of ketamine (e.g., inflammatory, metabolic, glutamatergic, and monoaminergic actions). For example, disturbances in the inflammation in the human central nervous system, and the associated glutamatergic dysregulation, are implicated as mechanistically relevant to mood disorders [46]). As ketamine has direct and indirect actions on glutamatergic signalling and inflammation, it is not surprising that pretreatment inflammatory and/or glutamatergic biomarkers may predict ketamine response.

The available evidence indicates that ketamine exerts rapid and robust antidepressant effects in patients with TRD. Herein, we identified several preliminary variables that are associated with a positive response to IV ketamine treatment. The observation that pretreatment inflammatory markers may have predictive potential coheres with other lines of evidence, indicating that a cross talk exists between inflammatory and glutamatergic systems [47]. Moreover, it has been further conjectured that individuals with disturbances in central amino acid neurotransmitters (i.e., GABA, glutamate) and/or pro-inflammatory cytokines (e.g., reward processing abnormalities) may particularly benefit from ketamine treatment. Ketamine has also been hypothesized to engage neuroplasticity processes; this observation would be in keeping with the moderational effects of the BDNF Val66Met SNP on the treatment response, as BDNF is known to be highly involved in neuroplastic processes within the brain.

Antidepressant treatment response is highly variable among patients, which may reflect the differences in the underlying pathophysiology, genetic architecture, clinical features, etc. With the identification of novel pretreatment predictors (i.e., phenotypic and biological variables) of response, there will be an opportunity for personalized healthcare and significantly improved clinical outcomes for patients. The absence of long-term clinical data, concerns related to the safety, and the tolerability of short- and long-term ketamine exposure, as well as the expense of IV administration invite the need for careful patient selection and treatment candidacy. Another important consideration is the effect size (i.e., predictive value) of individual pretreatment predictors, which would help to stratify patients for treatment accordingly.

The current review has several significant limitations. The most significant limitation was the small sample size of many of the studies that were included in the review. Another limitation is the heterogeneity of the study designs. Other limitations include lack of randomization, open-label trials, and lack of healthy control groups. Currently, no single pretreatment variable has been able to demonstrate a robust prediction for any antidepressant treatment response in MDD or BD. Instead, a combination of phenotypic (e.g., sociodemographic, clinical) and biological (e.g., neuroimaging, genetic) predictor variables will be necessary to identify patients who will be more likely to respond to ketamine. Endeavors are currently underway to identify the predictors of response to antidepressant treatment broadly, including, but not limited to, ketamine. It could be conjectured that machine learning techniques or capabilities may more likely be able to refine the phenotype that is most likely to benefit or not benefit from TRD therapy. It would be especially interesting to identify the subdomain response predictors e.g., improvements in cognitive measures and reward measures. Furthermore, there is a repository of studies evaluating ketamine studies on psychosocial and workplace function, consequently, identifying the predictors of response as defined by functional improvement and/or other patient reported outcomes (PROs) is an additional research vista. Previous epidemiological studies have found that the combination of having a history of unemployment and anticipated job insecurity increased the risk of developing depression [48]. Conventional antidepressants improve workplace functioning in patients that are suffering from MDD, and further studies on the effects of ketamine on workplace functioning is warranted [49].

## 5. Conclusions

Herein, we identified several putative pretreatments that may predict the antidepressant response to IV ketamine (see Table 1). Of the foregoing variables, the most replicated are BMI and FHP. A phenotypic response pattern to ketamine treatment for TRD is far from complete. Notwithstanding, disparate variables that are indicative of illness complexity emerge, with indirect evidence suggesting metabolic inflammatory alterations as pretreatment response prediction and cognitive impairment. Sophisticated data-driven computational methods that are iterative and agnostic are more likely to provide actionable baseline pretreatment prediction information.

## Figures and Tables

**Table 1:** Predictors of ketamine response.

Predictors of Ketamine Response		Studies	Number of Subjects
Sociodemographic	None	None	None
Clinical	Positive family history of alcohol use disorder in a first-degree relative (FHP)	Phelps et al. 2009 [17].	MDD (*n* = 26)
		Luckenbaugh et al. 2012 [18].	MDD and BD (*n* = 33)
		Niciu et al. 2014 [19].	MDD (*n* = 52)
	High BMI	Niciu et al. 2014 [19].	MDD (*n* = 108)
	No prior suicide history	Niciu et al. 2014 [19].	MDD (*n* = 108)
Peripheral biochemistry	Low baseline adiponectin levels	Machado-Vieira et al. 2017 [20].	MDD (*n* = 49) BD (*n* = 31)
	High baseline peripheral B12 level	Permoda-Osip et al. 2013 [21].	BD (*n* = 20)
Polysomnography	Low baseline delta sleep ratio	Duncan et al. 2013 [22].	MDD (*n* = 30)
Neurochemistry	Low Glx/glutamate ratio	Salvadore et al. 2012 [23].	MDD (*n* = 14)
Neuroimaging	Increased pretreatment anterior cingulate cortex activity	Salvadore et al. 2009 [24].	MDD (*n* = 11) HC (*n* = 11)
Genetics	Val66Met BDNF allele	Laje et al. 2012 [25].	MDD (*n* = 62)
Cognition	Poor baseline neurocognitive score	Murrough et al. 2013 [26].	MDD (*n* = 25)
